# The Effect of Changing the Toothbrush on the Marginal Gingiva Microcirculation in the Adolescent Population—A Laser Doppler Flowmetry Assessment

**DOI:** 10.3390/diagnostics12081830

**Published:** 2022-07-29

**Authors:** Mariana I. Miron, Madalina Barcutean, Ruxandra E. Luca, Carmen D. Todea, Anca Tudor, Emilia Ogodescu

**Affiliations:** 1 Department of Oral Rehabilitation and Dental Emergencies, Faculty of Dentistry, “Victor Babes” University of Medicine and Pharmacy, Eftimie Murgu Square No. 2, 300041 Timisoara, Romania; miron.mariana@umft.ro; 2 Interdisciplinary Research Center for Dental Medical Research, Lasers and Innovative Technologies, Revolutiei 1989 Avenue no. 9, 300070 Timisoara, Romania; 3 S.C. BEA OLAH DENT S.R.L., Geneva Avenue, No. 1, Ap. SAD No. 7, 300129 Timisoara, Romania; madalina27tm@yahoo.com; 4 Department of Functional Sciences, Faculty of Medicine, “Victor Babes” University of Medicine and Pharmacy, Eftimie Murgu Square No. 2, 300041 Timisoara, Romania; atudor@umft.ro; 5 Pediatric Dentistry Research Center, Department of Pediatric Dentistry, Faculty of Dentistry, “Victor Babes” University of Medicine and Pharmacy, Eftimie Murgu Square No. 2, 300041 Timisoara, Romania; ogodescu.emilia@umft.ro

**Keywords:** periodontal probing depth, gingival index, gingival blood flow, laser Doppler flowmetry

## Abstract

It is well-known that proper tooth brushing has the effect of stimulating microcirculation in a healthy gingiva. The aim of this study was to evaluate the microcirculation dynamics at the level of healthy marginal gingiva in adolescents after changing their toothbrush. Three evaluation instruments were employed to assess and quantify the effects on the marginal gingiva: the periodontal probing depth (PD), gingival index (GI) and laser Doppler flowmetry (LDF). A total of 12 adolescents, aged between 15 and 20, were enrolled in the study, resulting in a total of 72 frontal upper teeth for PD and GI and 48 gingival interdental sites for LDF assessment. For each measurement, the pulp blood flow signal was recorded for 1 min and represented as a pulsatory signal. Data were collected in four moments: before the toothbrush changed and 24 h, 7 days and 14 days after. For the statistical analysis, the nonparametric Friedman test was applied for comparisons between more than two pair numeric series and for comparisons between two sets of pair values without Gaussian distribution, the Wilcoxon signed-rank test was applied. The paired *t* test was used for two sets of normally distributed paired values. The results showed that using a new toothbrush in the adolescent population with healthy gingiva can induce statistically significant increases in gingival blood flow, which remain at a high level even at two weeks. The GI also increases significantly at 7 days and 14 days, while the periodontal PD does not change significantly.

## 1. Introduction

The toothbrush remains, without any doubt, one of the most important tools for maintaining proper oral hygiene. A correct brushing technique allows for good control of the bacterial plaque at the level of the dento-periodontal surfaces [1].

The effect of tooth brushing on gingival blood flow has been a topic of interest for researchers, being studied in both animals and human subjects. In 1981, Nuki et al. [2] performed a study on dogs and recorded the evolution of blood flow to the capillaries in the attached gingiva. For this purpose, they used measurements of the clearance of a diffusible isotope such as 133Xe, which are known to reflect changes in the blood flow rate in capillaries. Later, in 1997, Perry et al. [3], quantified the blood flow following tooth brushing using laser Doppler flowmetry (LDF) in the healthy gingiva of humans. LDF is an objective and noninvasive technique that allows for the measurement of microcirculation in human and animal tissues and records the blood flow continuously and in real time. Due to the well-represented microcirculation of the gingiva, this technique is used successfully in the evaluation of gingival vascular microdynamics, both in health and in disease [4,5,6,7,8,9].

Gingivitis is a defensive reaction to a harmful stimulus that can be of various etiologies (microbial, thermal, chemical, mechanical). Epidemiological studies show that gingivitis can occur at any age. Gingivitis of multifactorial etiology and in different stages can be identified in a large part of adolescents. Gum disease is usually caused by a dental plaque, an invisible sticky layer of germs that forms naturally on the teeth and gums and contains bacteria that make toxins that irritate and damage the gums. In the initial stage, gingivitis is quickly and completely reversible if the irritating factor is completely removed. It is recognized that vascular dynamics in the gingival tissues are different in healthy compared with disease states [10]. Healthy gums can also show significant variations in blood flow due to the actions of specific factors of varying etiology.

Adolescents are a special category of subjects, in a stage of development characterized by spectacular transformations in all aspects: somato-physiological, psychological (involving the intellect, memory, language, motivation, affectivity, morality) and social [11,12,13]. At this age, influenced by hormonal evolution, young people show particular behaviors related to food hygiene, general and oral hygiene [14], sleep–wake rhythm, clothing and physical appearance, smoking, drug addictions and alcoholism. In addition, teenagers attach great importance to the aesthetics of the smile, being influenced by the models the media promote who have ideal beauty including a bright smile.

Adolescents, due to the particularities of psycho-somatic development and the behavior of adapting to the social group, represent a category of patients in whom the marginal periodontium is subjected to the action of several stressors both general and local [15]. Moreover, adolescents tend to consume more snacks and beverages between meals. In this particular context, some factors may cause changes in the response of the periodontium in an attempt to maintain gingival health [16]. All of these predisposing factors increase the risk of dental lesions and early inflammatory gingival disease in adolescents [14,17,18], and the most common method of controlling oral hygiene is tooth brushing, i.e., individual oral prophylaxis with interdental brushes for adolescents with fixed orthodontic treatment [19], and local fluorization. However, the efficiency of toothbrushing is influenced by certain factors such as the brushing technique, the frequency and duration of brushing, the type and shape of the toothbrush and the type of toothpaste used [20]. Currently, the best known brushing techniques are the Bass technique, the modified Bass technique, the Stillmann technique, the modified Stillmann technique, the Fones technique and the Charters technique [21].

Some authors recommend changing the toothbrush after 3–4 weeks, as bacterial contamination of the toothbrush increases significantly after 3 months of use compared with toothbrushes used for only one month [22,23]. In addition, out of a desire to improve their appearance, adolescents may try to change their toothbrush more often. The diversity of toothbrushes and toothpastes available on the market, as well as the lack of correct specialist information, could be confusing for teenagers. Therefore, young people need the advice of their dentist to choose the right type of toothbrush and the right brushing technique, as well as the appropriate time period to change the toothbrush, to maintain their periodontal health and achieve the desired aesthetic appearance [24,25].

However, we can self-induce gingivitis; for example if we brush too hard or aggressively or use a toothbrush with hard bristles, we can damage the gingival mucosa, inducing pain and inflammation. In this context, we can ask ourselves if the more frequent change of the toothbrush, faster than a month, can represent a factor of stimulation or local microirritation. The aim of this study was to evaluate the microcirculation in healthy marginal gingiva in adolescents after changing the toothbrush using laser Doppler flowmetry. The examination of the marginal gingiva also included two classic periodontal tests, the periodontal probing depth and the Löe–Silness gingival index. A statistical/null hypothesis was formulated that there are no statistically significant differences between gingival blood flow recorded before changing the toothbrush and that recorded after using a new toothbrush.

## 2. Materials and Methods

### 2.1. Sample Selection

The study was performed at the Municipal Hospital of Timisoara, Romania, affiliated with the Victor Babes University of Medicine and Pharmacy in Timisoara. The study included twelve adolescent volunteers selected from among the Oral Rehabilitation and Emergencies in Dentistry Discipline patients. The subjects were in good general health, nonsmokers who presented all frontal teeth with a healthy gingiva (following clinical examination). All the participants were informed about the purpose of the study and the work protocol, and they gave their written consent. In the case of minor adolescents, the consent of their parents was obtained. The study was carried out in accordance with the Declaration of Helsinki, and it was approved by the Ethics Committee of the Municipal Hospital (Approval No E-2815/19 May 2022).

### 2.2. Evaluating the Gingival Response

**Figure 1:**
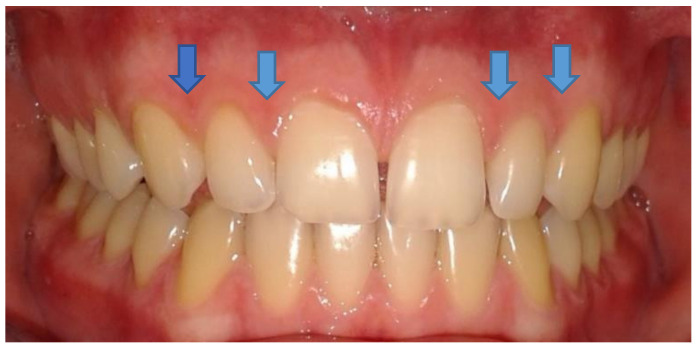
The sites for assessment of marginal gingival microcirculation using laser Doppler flowmetry are indicated by blue arrows.

Three evaluation instruments were employed to assess and quantify the effects on the marginal gingiva as a result of the use of a new toothbrush:

(i) Periodontal probing was performed to determine the depth of the gingival sulcus and the presence or absence of periodontal attachment loss. The periodontal probing depth is the distance between the free gingival margin and the base of the sulcus (or the base of the periodontal pocket, if there is a loss of attachment) [21]. A periodontal calibrated probe UNC 15 (Dentech, Debrecen, Hungary, DB code 1768) was used for the periodontal probing. The probe has 15 gradations of 1 mm each, and the gradations at 5 mm, 10 mm and 15 mm are marked with a black band. The periodontal examination was performed in six points per tooth: mesio-vestibular, centro-vestibular, disto-vestibular, mesio-lingual, centro-lingual and disto-lingual.

(ii) The gingival index (Löe and Silness) was calculated to assess the gingival condition and to record qualitative changes in the gingiva, being the most widely used index for assessing the severity of gingival inflammation. It scores the marginal and interproximal tissues separately on the basis of 0 to 3. The presence of inflammation and gingival bleeding is determined in four points for each tooth: vestibular, lingual, mesial and distal at the level of the marginal gingiva. Data collection is performed by periodontal probing, passing the periodontal probe along the gingival wall of the gingival sulcus. Depending on the data recorded, each area examined receives a score from 0 to 3, as follows: 0 = normal appearance of the gum; 1 = slight inflammation—discreet discoloration, slight edema, no bleeding on probing; 2 = moderate inflammation—erythema, edema, shiny appearance of the gums, the presence of bleeding on probing; 3 = severe inflammation—severe erythema and edema, ulceration, tendency to spontaneous gingival bleeding. The individual gingival index of the tooth represents the arithmetic mean of the four scores. The patient’s final gingival index results from the sum of the individual scores for each tooth divided by the total number of teeth examined. A score between 0.1 and 1.0 is mild inflammation, 1.1–2.0 is moderate inflammation and 2.1–3.0 is severe inflammation.

(iii) Laser Doppler flowmetry (LDF) was performed for recording the vascular micro-dynamics at the gingival level. To assess the therapeutic response with LDF, the equipment comprised the following: a MoorLab laser Doppler device for general medical use (Laser Doppler MoorLab instrument VMS-LDF2, Moor Instruments Ltd., Axminster, UK); straight optic probe VP3, with a length of 10 mm, built to be used on the oral mucosa/teeth and allows the recording of blood flow from the gingival superficial capillaries, expressed in relative flow units in the range 0–1000 PU (perfusion units), with an accuracy of ± 10 PU and ±3%. The laser Doppler signal acquisition was performed according to our previous studies [26,27,28]. In order to stabilize the laser probe in the selected area on the gingiva (Figure 1), a double silicone impression was taken using Kit Optosil Comfort Putty and Xantopren Comfort Light, Haereus (Heraeus Kulzer, GmbH Leipziger Straße 2, Hanau, Germany).

**Figure 2:**
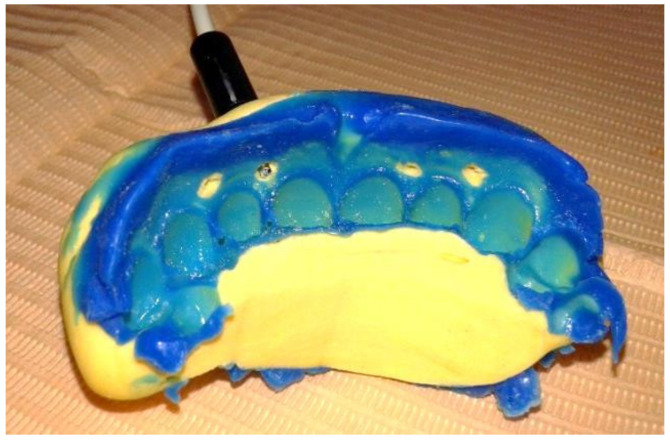
The probe holder, used for positioning the LDF probe (mucosal view).

This is a material based on condensing silicone, which allows for making an impression with high dimensional stability in moist dental environments, thus allowing precise reproduction detail (Figure 2). This impression was further used as an LDF probe holder for acquiring laser Doppler signals.

### 2.3. Sample Description and Study Protocol

A total of 12 adolescents, aged between 15 and 20 (6 female and 6 male) were enrolled in the study from May 2022 to June 2022, resulting in a total of 72 teeth for periodontal probing and gingival index, and 48 interdental sites for laser Doppler flowmetry assessment. The inclusion criteria were as follows: (1) voluntary participation, (2) adolescent subjects (aged between 15 and 20) (3) who present intact maxillary frontal teeth, (4) who had healthy marginal gingiva (periodontal probing depth at most equal to 3 mm); (5) who did not change their toothbrush in the last 4 weeks before participating in the study; (6) and who had healthy general status. Exclusion criteria: (1) subjects with periodontal disease or undergoing periodontal treatment; (2) with soft tissue damage; (3) undergoing orthodontic treatment; (4) with dento-maxillary anomalies; (5) with an infection in the oral cavity, (6) with diabetes or other general conditions that involve long-term drug treatment or (7) with addictions or (8) who had undergone chemotherapy or radiation therapy in the past year

The sample size was identified with a power of 0.90 and a significance level of 0.05 for 12 subjects (with 6 measurements each). A G Power test was performed for Wilcoxon signed-rank family tests (matched pairs) with two tails and a normal parent distribution, 95% power, 0.05 level of significance and 0.44 as an allocation ratio.

#### Working Protocol

Before collecting the data, the subjects signed the informed consent, completed a medical questionnaire, an oral hygiene questionnaire, and the periodontal record. Intraoral photographs were also taken and the probe holder (a double silicone impression) constructed.

The subjects were instructed on maintaining good oral hygiene and a healthy diet, and they were asked not to use dental floss, anti-inflammatories, or professional brushing and scaling throughout the study. During the study, all subjects received and used the same toothbrush (Colgate Extra Clean medium) and the same toothpaste (Colgate Cavity Protection). In addition, they were instructed about the brushing technique that they would have to use during the study, the modified Bass technique. Brushing was to be performed twice a day, in the morning and in the evening, for 3 min, and all assessments were carried out in the afternoon by the same dentist. Subjects were also instructed not to eat or to brush their teeth at least two hours before the determinations. According to Gleissner et al. [10], gingival blood flow to the healthy gingiva returns to normal after 60 min.

The teeth evaluated in the study were the frontals of the upper jaw (1.3., 1.2., 1.1., 2.1., 2.2. and 2.3.), and the periodontal probing was performed by the same evaluator: Using light force, the periodontal probe was inserted in the gingival sulcus in contact with the dental surface in the long axis of the tooth. The periodontal examination was performed in six points per tooth, already mentioned. The LDF test sites are located on the vestibular face of the interdental papillae at the level of the maxillary front teeth, that is: the papilla between 1.3. and 1.2., between 1.2. and 1.1., between 2.1. and 2.2. and between 2.2. and 2.3. (Figure 1). For each measurement, the pulp blood flow signal was recorded for 1 min and represented as a pulsatory signal. The flow is related to the product of the average speed and concentration of mobile red blood cells in the tissue sample volume. The digital signal’s means and standard deviations were recorded for further analysis.

Data were collected in four stages: stage I—before changing the toothbrush; stage II—24 h after brushing with a new toothbrush; stage III—7 days after changing the toothbrush and stage IV—14 days after changing the toothbrush.

### 2.4. Data Analysis

In this prospective analytical study, the collection of data from the independent sample was performed in four distinct moments. The variables studied were the periodontal probing depth, gingival index and gingival blood flow (from the vestibular area of the interdental papilla), corresponding to the upper frontals. Data collection was performed by periodontal probing, gingival index determination (according to the protocol described above) and laser Doppler flowmetry, respectively. The collected data are quantitative and numerical: probing depth (mm), gingival index (score calculated using quantitative data) and mean gingival blood flow (arbitrary perfusion units—PU).

Statistical data were processed using IBM SPSS v17.0 software. Descriptive statistics were performed for the analysis of numerical variables (minimum and maximum values, mean + standard deviation for normally distributed values and median with interquartile range for values without Gaussian distribution). The nonparametric Friedman test was applied for comparisons between more than two pairs of numeric series, and for comparisons between two sets of pair values without Gaussian distribution, the Wilcoxon signed-rank test was applied. The paired *t* test was used for two sets of normally distributed paired values. The results were considered significant at *p* <0.01.

## 3. Results

Table S1 (Supplementary Materials) shows the gingival indices recorded for the 6 maxillary frontal teeth included in the study. For the gingival index, descriptive statistics are presented in Table 1.

Comparisons between two-by-two moments were made with the nonparametric Wilcoxon signed-rank test, and the results are presented in Table 2.

Table S2 (Supplementary Materials) shows the mean periodontal probing depths for the six maxillary frontal teeth included in the study. For the periodontal probing depth, descriptive statistics are presented in Table 3.

The differences between the values of the probing depth recorded in the four time moments are not statistically significant (Friedman test, *p* = 0.203). Comparisons between two-by-two time moments were made with the Wilcoxon signed-rank nonparametric test, and the results were not statistically significant (Table 4).

**Figure 3:**
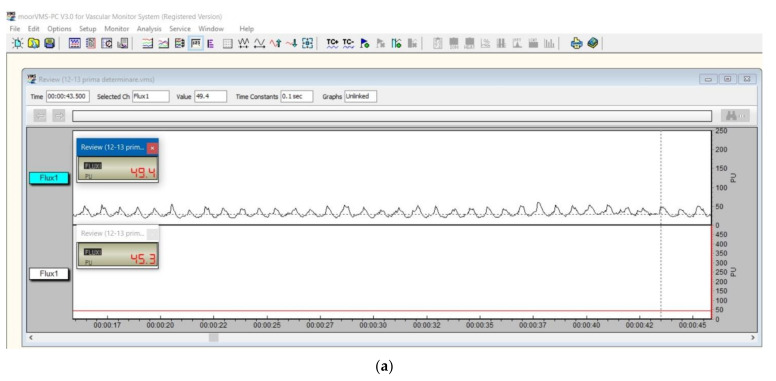

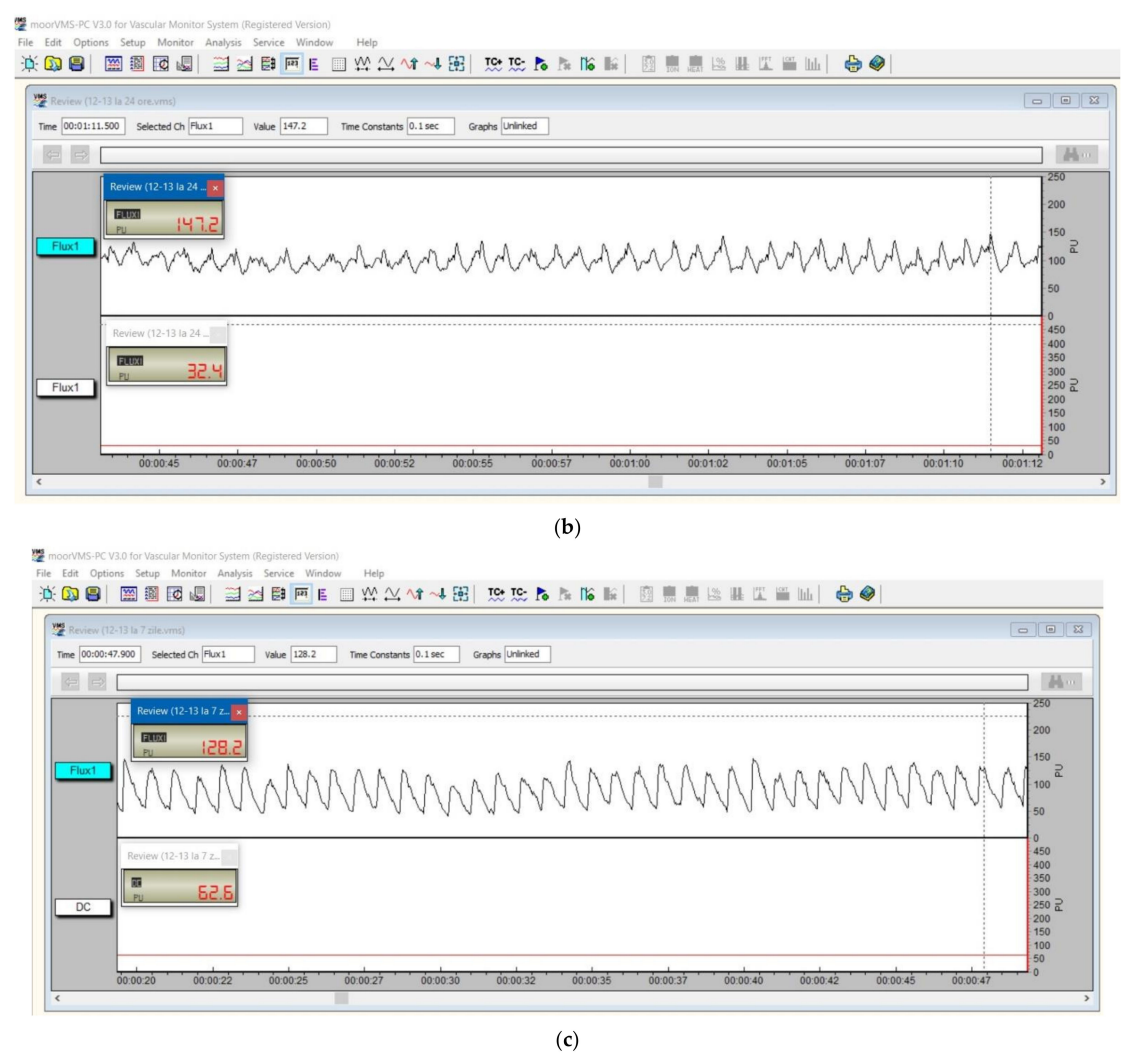

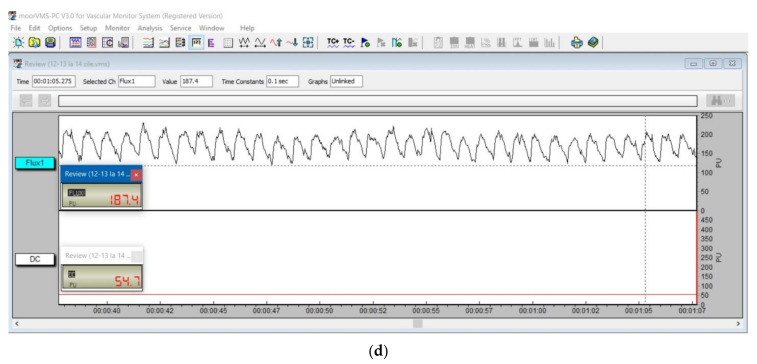
Laser Doppler gingival signal for 12–13 interdental papilla: (**a**) initial and (**b**) 24 h; (**c**) 7 days and (**d**) 14 days after using a new toothbrush. The pulsatory signal is shown in the upper window.

Vascular micro-dynamics at the gingiva level were measured as pulsatory signals and recorded in the four time moments of the study (Figure 3).

Table S3 (Supplementary Materials) shows the actual laser Doppler flowmetry measurements for the 48 gingival interdental papillae.

For the mean gingival blood flow, registered for the interdental papillae considered in the study, the descriptive statistics are presented in Table 5.

Comparisons between mean gingival blood flow, recorded in the four moments of determination were made with the nonparametric Wilcoxon signed-rank test (Table 6).

In Table 7, we can see the results of gingival blood flow obtained after the comparison of two-by-two moments of determination, using paired *t* Test (*p* < 0.001).

**Figure 4:**
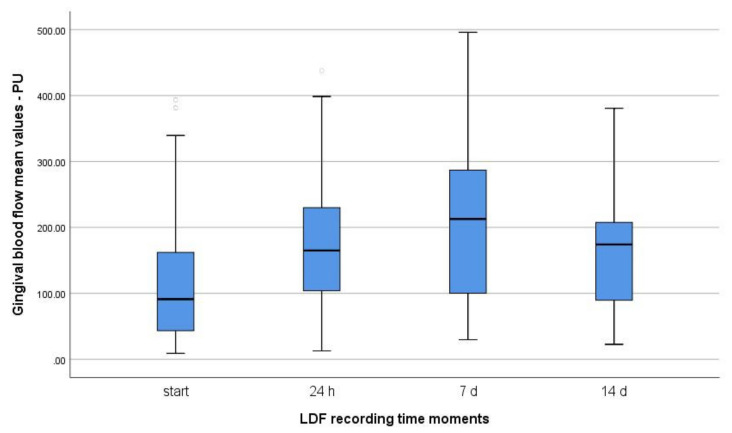
Comparative representation of the mean gingival blood flow in the four evaluation moments considered in the study.

Figure 4 shows the evolution of gingival blood flow in the four moments of determination: before changing the toothbrush and 24 h; 7 days and 14 days after brushing with a new toothbrush.

## 4. Discussion

In the present study we intended to observe whether brushing with a new toothbrush, a daily and mandatory behavior, can produce significant changes in the vascular microdynamics of the marginal gingiva of adolescents. In addition to classical tests, periodontal probing depth and gingival index, we used laser Doppler flowmetry, knowing that this is a noninvasive, objective method that can identify changes in blood flow after brushing [2,3] in real time and before the occurrence of clinical signs.

The results obtained following our study allowed for the rejection of the statistical/null hypothesis that there would be no statistically significant differences between gingival blood flow recorded before changing the toothbrush and that recorded after using a new toothbrush.

According to our results (Table 6), after changing the toothbrush, the blood flow from the interdental marginal gingiva increased statistically significantly at 24 h, 7 days and 14 days from the initial flow (Wilcoxon signed-rank test, *p* < 0.001). If we compare the two-by-two moments of determination, it is observed that the gingival blood flow increases significantly at 7 days compared with 24 h (paired *t* test, *p* < 0.001). On the other hand, the blood flow recorded at 14 days decreased and was not statistically significant from 24 h (paired *t* test, *p* = 0.802) but decreased and statistically significant compared with 7 days (paired *t* test, *p* < 0.001) (Table 7). Our results, obtained at 24 h are consistent with those of other studies in which it was observed that tooth brushing has an effect of the immediate stimulation of gingival blood flow. Perry et al. [3] quantified the blood flow following tooth brushing using laser Doppler flowmetry (LDF), in the healthy gingiva of humans. They found that tooth brushing for both 3 and 10 s significantly increased gingival blood flow in the papillary gingiva of healthy individuals. Furthermore, they found that the longer brushing time did not result in greater blood flow effects. It would appear that stimulatory effects to the microcirculation occur quickly and are limited in the extent of the increase, probably due to the physiologic capacity of the microcirculation.

In another interesting study, Wada-Takahashi et al. [29] aimed to investigate changes in the gingival microcirculation of Wistar rats after gingival massage using a a laser Doppler flowmeter. They considered that gingival massage has an effect on the gingiva, most likely as a result of the activation of eNOS due to the shearing stress applied during gingival massage and the resulting increase in NO, which triggers a vascular relaxation response. Their study investigated reactive hyperemia as an endothelium-dependent vasorelaxation response and indicator of vascular endothelial cell function and peripheral microcirculation adjustment function. Wada-Takahashi et al. concluded, using laser Doppler flowmetry, that physical stimulation (gingival massage) improves gingival microcirculatory function and morphology and is effective for maintaining oral health. These consistent findings represent a valuable contribution regarding the assessment of gingival microcirculation using laser Doppler flowmetry.

However, in the case of the present study, analyzing the evolution of the mean gingival blood flow recorded in the four evaluation moments considered in the study, it can be seen that there was an increase in gingival blood flow at 24 h and after 7 days that maintained at relatively high values at 14 days as well; in fact, the average gingival blood flow was even higher at 14 days than at 7 days (Figure 4). Our results differ from those of Gleissner et al. [10], who determined that gingival blood flow in the healthy gingiva, returns to normal after 60 min; however, they did not use a new toothbrush.

Related to Gingival Index, our results (Table 2) showed that the GI increases statistically significant at 24 h from baseline (Wilcoxon signed-rank test, *p* < 0.001). The gingival index also increases significantly at 7 days and 14 days from baseline (Wilcoxon signed-rank test, *p* < 0.00. However, at 14 days the GI decreases insignificantly compared with the value at 7 days (*p* = 0.039). Thus, the results of the present study showed a statistically significant increase in gingival indices after changing the toothbrush. Tangade et al. [30] showed that the use of new toothbrushes leads decreases plaque index and improves gingival indices, while Asadoorian et al. [31] said that new toothbrushes lead to a decrease in plaque index but worn toothbrushes lead to an improvement in gingival indices.

The gingival index reflects the clinical features of the marginal periodontium: discoloration, edema, the presence or absence of bleeding on probing. The fact that this index increases significantly at 7 days and 14 days from the initial moment may suggest the presence of a microirritation in the surface capillaries produced by the use of a new toothbrush with medium-hard bristles. Even so, the gingival index decreases at 14 days compared with 7 days, which could indicate the intervention of the adaptive mechanisms at the level of the capillaries. Another explanation may be related to the erosion of the toothbrush: McLey [32] proved that after 30 min of use, a manual toothbrush with soft bristles will have the tips of worn bristles, with a flattened shape with rounded edges regardless of the initial shape of the tip of the bristles. A period of 30 min of use is equivalent to 10 sessions of use of 3 min each. In our study, subjects were recommended to brush their teeth twice a day, in the morning and in the evening. The recommended brushing time was 3 min. Thus, 10 sessions of using the toothbrush take place in the first 5 days. If the toothbrushes in our study were soft bristles, the brushes would have worn out after 5 days of use. But the toothbrushes received by the subjects in our study were of medium hardness, so we can assume that rounding the tips of the brushes took more than 5 days with the toothbrush in our study.

The results for periodontal probing depth showed that the differences between the values of the probing depth recorded in the four time moments are not statistically significant (Friedman test, *p* = 0.203). Comparisons between two-by-two time moments were made with the Wilcoxon signed-rank nonparametric test, and the results obtained showed that there are no statistically significant differences between the probing depths regardless of the paired moments that were compared (Table 4). The periodontal probing depths at 14 days are insignificantly lower than the initial ones (Wilcoxon signed-rank test, *p* = 0.015) and those at 7 days (*p* = 0.056).

There was no significant increase in probing depth during the 14 days, which means that there was no increase in the gingival volume or loss of attachment, which was to be expected in subjects with healthy gingiva. The mean periodontal probing depth was 1.67 mm initially and 1.64 mm at 14 days after changing the toothbrush. The data obtained from this study are in line with those described by Perry [3], who observed that gingival health was present in both the initial session and the one-month session; average periodontal probing depth was 2.88 mm initially and 2.97 mm one month after changing the toothbrush.

However, there are some particular aspects that need to be considered in this study. laser Doppler flowmetry (LDF) allows for measurements of microcirculation in the tissues of humans and animals and records blood flow in about 1 mm^3^ of tissue but measurements of microcirculation can be made with low reliability. The main disadvantage of LDF is that it does not accurately measure blood flow, so it cannot be used to calculate absolute blood flow (e.g., in units of mL/min/100 g tissue); instead, LDF produces only relative blood flow [4,33].

In the results of the present study, immediately after changing the toothbrush and even after the first 14 days, high gingival blood flows were obtained, which can be clinically interpreted as a local stimulation of the gingival circulation.

Although the other two clinical parameters improved after changing the toothbrush, LDF (as an objective and noninvasive diagnostic tool) shows in real time that at the preclinical level, there is local stimulation of microcirculation. Taking into account the fragile structure of the gingival tissue, especially in adolescents, this biostimulation effect in continuous form, can exceed the capacity of local vascular adaptation and produce local stasis, with the appearance of inflammatory infiltrate. Additional studies are needed to show how long after the change of the toothbrush the microcirculation of the marginal gum is normalized and when the vascular adaptive capacity is eventually exceeded. Changing the toothbrush after two weeks may be at some point a microirritation factor for local gingivitis. Maintaining gingival microirritation can eventually lead to inflammation of the marginal and even the supporting periodontium.

In addition, future studies with representative samples in terms of structure and size are needed, together with a standardization of the tooth brushing technique, including the pressure exerted by the subject on the toothbrush during brushing. Moreover, the age range of adolescents was quite wide, taking into account the fact that during this period, there are many and varied variables, both physiological and behavioral, that can influence gingival status.

Furthermore, evaluating of vascular microdynamics at the level of the marginal gingiva when using one-, two-, or three-month-old toothbrushes is a relevant and appropriate research direction that must be conduct in a future study.

## 5. Conclusions

According to the results of the present study, changing the toothbrush with a new one of medium hardness in the adolescent population with healthy gingiva can induce statistically significant increases in gingival blood flow, which remain at a high level even at two weeks. The gingival index also increases significantly at 7 days and 14 days from the first time of using a new toothbrush, while the periodontal probing depth does not change significantly.

As a general observation of this study, the analysis of the obtained results indicates that changing the toothbrush at less than a month could be a factor in gingival microirritation in the adolescent population with healthy gingiva.

In conclusion, based on the results of this study and taking into account the limitations of this study, we recommend that adolescents do not change their toothbrush sooner than a month even if using a correct toothbrushing technique.

## Figures and Tables

**Table 1:** Descriptive statistics for gingival index (GI).

			Descriptive Statistics for Gingival Index				
GI	n	Mean	Std. Deviation	Median	Interquartile Range	Minimum	Maximum
GI initial	72	0.319	0.326	0.25	0.5	0	1
GI 24 h	72	0.493	0.333	0.5	0.5	0	1
GI 7 days	72	1.021	0.161	1	0	0.75	1.50
GI 14 days	72	0.990	0.142	1	0	0.75	1.25

Because GI did not have a normal distribution (Shapiro–Wilk test, *p* < 0.001), comparisons between gingival indices at the four time points were made with the nonparametric Friedman test. The differences in the gingival index between the four time points were statistically significant (*p* < 0.001).

**Table 2:** Wilcoxon signed-rank test results for gingival index (GI).

Wilcoxon Signed-Rank Test			
Pairs	df	Z	Asymp. Sig. (2-Tailed)
GI 24—GI initial	72	−5.056 ^a^	<0.001 *
GI 7 days—GI initial	72	−7.197 ^a^	<0.001 *
GI 7 days—GI 24 h	72	−6.941 ^a^	<0.001 *
GI 14 days—GI initial	72	−7.296 ^a^	<0.001 *
GI 14 days—GI 24 h	72	−7.050 ^a^	<0.001 *
GI 14 days—GI 7 days	72	−2.065 ^a^	0.039

^a^ Based on negative ranks. *—significant differences.

**Table 3:** Descriptive statistics for probing depth (PD).

			Descriptive Statistics for Probing Depth				
PD	n	Mean	Std. Deviation	Median	Interquartile Range	Minimum	Maximum
PD initial	72	1.679	0.326	1.66	0.17	1.00	2.33
PD 24 h	72	1.671	0.333	1.66	0.17	1.00	2.33
PD 7 days	72	1.674	0.161	1.66	0.17	1.00	2.33
PD 14 days	72	1.643	0.142	1.66	0	1.00	2.33

**Table 4:** Wilcoxon signed-rank test results for probing depth (PD).

Wilcoxon Signed Ranks Test			
Pairs	df	Z	Asymp. Sig. (2-Tailed)
PD 24—PD initial	72	−0.991 ^a^	0.322
PD 7 days—PD initial	72	−0.691 ^a^	0.489
PD 7 days—PD 24 h	72	−0.399 ^a^	0.690
PD 14 days—PD initial	72	−2.426 ^a^	0.015
PD 14 days—PD 24 h	72	−1.504 ^a^	0.133
PD 14 days—PD 7 days	72	−1.907 ^a^	0.056

^a^ Based on negative ranks.

**Table 5:** Descriptive statistics for gingival blood flow (BF).

Descriptive Statistics for Gingival Blood Flow					
BF	n	Mean ± Std. Dev.	Median (Interquartile Range)	Minimum	Maximum
BF initial	48	116.4 ± 98.24	91.25 (120.6)	8.9	393.6
BF 24 h	48	159.2 ± 102.49	151.35 (142.7)	8.9	437.6
BF 7 days	48	206.5 ± 113.78	212.8 (190.3)	29.6	495.9
BF 14 days	48	157.2 ± 88.35	170.15 (123.2)	10.9	380.8

The initial blood flow means do not have a normal distribution (Shapiro–Wilk Test, *p* < 0.001), so the comparison between the mean blood flows at the four time points was made with the nonparametric Friedman test. The differences between the mean gingival blood flow recorded at the four time points were statistically significant (Friedman test, *p* < 0.001).

**Table 6:** Comparisons between BF initial (without normal distribution) and the other moments of time for gingival blood flow recorded with LDF.

Wilcoxon Signed Ranks Test			
Pairs	df	Z	Asymp. Sig. (2-Tailed)
BF 24—BF initial	48	−4.523 ^a^	<0.001 *
BF 7 days—BF initial	48	−5.436 ^a^	<0.001 *
BF 14 days—BF initial	48	−3.790 ^a^	<0.001 *

^a^ Based on negative ranks. *—significant differences.

**Table 7:** Comparisons between two-by-two moments of time (with normal distribution) for gingival blood flow recorded with LDF.

Paired-Samples Test									
		Paired Differences					*t*	df	Sig. (2-tailed)
		Mean	Std. Dev.	Std. Error Mean	95% Confidence Interval of the Difference				
					Lower	Upper			
Pair 1	BF 24 h–7 d	−47.22	60.64	8.75	−64.83	−29.61	−5.40	48	<0.001 *
Pair 2	BF 24 h–14 d	2.06	56.45	8.15	−14.34	18.45	0.25	48	0.802
Pair 3	BF 7 d–14 d	49.28	73.78	10.65	27.85	70.70	4.63	48	<0.001 *

*—significant differences.

## Data Availability

Data can be requested from the corresponding authors upon reasonable request.

## Supplementary Materials

The following supporting information can be downloaded at: https://www.mdpi.com/article/10.3390/diagnostics12081830/s1, Table S1: Mean values of the periodontal probing depth at the level of the 6 maxillary frontal teeth included in study. Table S2: Gingival index scores recorded at the level of the 6 maxillary frontal teeth included in study. Table S3: The evolution of the mean values of the gingival blood flow (perfusion units—PU) registered at the level of the four evaluation sites (interdental papilla), in the four testing moments, considered in the study.

## Abbreviations

LDF	Laser Doppler Flowmetry
PU	Perfusion Units
BF	Blood Flow
PD	Probing Depth
GI	Gingival Index
