# Supplementary material for: The Effect of Changing the Toothbrush on the Marginal Gingiva Microcirculation in the Adolescent Population—A Laser Doppler Flowmetry Assessment
Source: Diagnostics (Basel). 2022 Jul 29;12(8):1830. doi: 10.3390/diagnostics12081830 (PMC9406728; doi:10.3390/diagnostics12081830)
Supplement: Supplementary file 1 [file diagnostics-12-01830-s001.zip › diagnostics-1799238-supplementary.pdf]

Table S1. Mean values of the periodontal probing depth at the level of the 6 maxillary frontal teeth included in study.

Patient	Tooth	Mean values for Periodontal Depth Probing (mm)			
		Initial	After 24 h	After 7 d	After 14 d
1	13	1.83	1.83	1.83	1.83
	12	2	1.83	1.66	1.83
	11	1.83	2	2	1.83
	21	1.83	2.16	1.66	1.66
	22	1.83	2	1.83	1.66
	23	2	2.16	1.66	1.83
2	13	2	1.83	1.83	1.83
	12	1.66	1.66	1.66	1.66
	11	1.66	1.33	1.66	1.66
	21	1.66	1.33	1.33	1.33
	22	1.5	1.5	1.5	1.5
	23	1.5	1.5	1.5	1.5
3	13	1.83	1.66	1.66	1.66
	12	1.83	1.66	1.83	1.66
	11	1.66	1.66	1.83	1.66
	21	1.66	1.66	1.66	1.66
	22	2	1.66	2	1.5
	23	1.83	1.66	1.83	1.66
4	13	1.66	1.66	1.66	1.66
	12	1.66	1.66	1.66	1.66
	11	1.66	1.66	1.66	1.66
	21	1.66	1.66	1.66	1.66
	22	1.66	1.66	1.66	1.66
	23	1.66	1.66	1.66	1.66
5	13	1.66	1.66	1.83	1.66
	12	1.66	1.66	1.66	1.66
	11	1.83	1.83	1.83	1.66
	21	1.66	1.66	1.66	1.66
	22	1.66	1.66	1.83	1.66
	23	1.66	1.66	1.66	2
6	13	1.83	2	2	1.66
	12	1.66	1.66	1.66	1.66
	11	1.83	1.66	1.66	1.66
	21	1.66	1.66	1.66	1.66
	22	1.83	1.66	1.66	1.66
	23	2	2	2	1.66
7	13	1.66	1.66	1.66	1.66
	12	1.66	1.66	1.66	1.66
	11	1.66	1.66	1.66	1.66
	21	1.66	1.66	1.66	1.66
	22	1.66	1.66	1.66	1.66
	23	1.83	1.66	1.83	2
8	13	2	2	2	2
	12	1.66	1.66	1.66	1.66
	11	1.66	1.66	1.66	1.66
	21	1.66	1.66	1.66	1.66
	22	1.66	1.66	1.66	1.66
	23	1.66	1.66	1.66	1.66
9	13	2.16	2.33	2.33	2.16
	12	2.33	2.33	2.33	2.33
	11	2	2.16	2.16	2
	21	2.16	2.16	2.16	2.16
	22	1.83	1.83	1.83	1.83
	23	2	2.16	2.16	2
10	13	1.66	1.83	1.66	1.66

	12	1.66	1.66	1.66	1.66
	11	1.66	1.66	1.66	1.66
	21	1.66	1.66	1.66	1.66
	22	1.83	1.83	1.83	2
	23	1.83	1.83	1.83	1.66
11	13	1.33	1.33	1.33	1.33
	12	1.33	1.33	1.33	1.33
	11	1.33	1.33	1.33	1.33
	21	1.33	1.33	1.33	1.33
	22	1.33	1.33	1.33	1.33
	23	1.33	1.33	1.33	1.33
12	13	1.33	1.33	1.33	1.33
	12	1	1	1	1
	11	1	1	1	1
	21	1	1	1	1
	22	1	1	1	1
	23	1.33	1.5	1.5	1.5

Table S2. Gingival index scores recorded at the level of the 6 maxillary frontal teeth included in study.

Patient	Tooth	Gingival Index Scores			
		Initial	After 24 h	After 7 d	After 14 d
1	13	0	0.25	1	1
	12	0.5	0.5	1	1
	11	0	0.5	1	1
	21	0	1	1	1
	22	1	1	1	1
	23	0	0.75	1	1
2	13	0.25	0.25	1	1
	12	0.25	0.5	1	1
	11	0	0	0.75	0.75
	21	0	0	1	1
	22	0.5	0.75	1.25	1.25
	23	0.25	0.5	1	1
3	13	1	1	1	1.25
	12	0.75	1	1	1.25
	11	0	0	1	1.25
	21	0	0	1	1
	22	1	1	1	1.25
	23	1	1	1	1
4	13	0.5	1	1	1
	12	0.5	0.5	1	1
	11	0.5	0.5	1	1
	21	0	0	1	0.75
	22	0	0	1	0.75
	23	0.5	0.5	1	1
5	13	0.5	1	1.5	1.25
	12	0.5	0.5	1.25	1.25
	11	0	0	1.25	1
	21	0	0	1.25	1
	22	0.5	0.5	1.5	1
	23	0.5	0.5	1.25	1
6	13	0.5	1	1	1
	12	0.5	0.5	1	1
	11	0	0	1	1
	21	0	0	1	0.75
	22	0.5	0.5	1	0.75
	23	0.5	0.5	1	1
7	13	0	0.25	1	1

	12	0.5	0.5	1	1
	11	0	0.25	1	1
	21	0	0.25	1	1
	22	1	0.75	1	1
	23	0	0.25	1	1
8	13	0.5	0.5	1.25	1
	12	0	0.5	1	1
	11	0	0.25	1	1
	21	0	0	0.75	0.75
	22	0.75	1	1.25	1.25
	23	0	0.5	1	1
9	13	0.75	1	1.25	1.25
	12	0.5	0.75	1	1
	11	0.25	0.75	0.75	0.75
	21	0.5	0.75	1	1
	22	0.75	1	1	1
	23	1	1	1.5	1.25
10	13	0	0.5	1	1
	12	0	0.5	0.75	0.75
	11	0.25	0.25	0.75	0.75
	21	0.25	0.25	0.75	0.75
	22	0.25	0.5	1	1
	23	0	0.5	1	1
11	13	0.5	0.5	1	1
	12	0.25	0.5	1	1
	11	0	0	0.75	0.75
	21	0.25	0.25	1	1
	22	0.5	0.5	1	1
	23	0.5	1	1.25	1
12	13	0.5	0.5	1	1
	12	0	0.5	1	1
	11	0	0	0.75	0.75
	21	0	0.25	0.75	0.75
	22	0	0.5	1	1
	23	0.75	0.75	1	1

Table S3. The evolution of the mean values of the gingival blood flow (perfusion units—PU) registered at the level of the four evaluation sites (interdental papilla), in the four testing moments, considered in the study.

Patient	Interdental Papilla	Initial		After 24 hours		After 7 days		After 14 days	
		Mean Values of the Gingival Blood Flow (PU)	Standard Deviation σ	Mean Values of the Gingival Blood Flow (PU)	Standard Deviation σ	Mean Values of the Gingival Blood Flow (PU)	Standard Deviation σ	Mean Values of the Gingival Blood Flow (PU)	Standard Deviation σ
1	*1.1.-1.2.*	11.6	2.5	8.9	2.3	77.5	12.2	30.9	7
	1.2.-1.3.	17.8	5.3	30.1	9.9	29.6	10.2	10.9	3.8
	2.1.-2.2.	8.9	1.2	13.8	4.8	38.5	8.1	22.7	7.8
	2.2.-2.3.	15.9	5.2	12.7	3.6	61.4	21.9	23.5	7.3
2	*1.1.-1.2.*	54.5	13.2	265.9	38.5	98.1	15.4	190.8	44.7
	1.2.-1.3.	39	10.5	98.5	16.5	92.8	30.2	178.3	30.7
	2.1.-2.2.	41.4	16.2	279.3	43.5	235.1	51.6	308.1	72.6
	2.2.-2.3.	45.3	14.2	265.7	61.4	281.7	56.9	119.2	21.3
3	*1.1.-1.2.*	234.4	45.9	237	50.5	244.5	50.5	220.2	44.7
	1.2.-1.3.	176.9	53.5	167.3	70.4	323.2	71.4	177.8	28.4
	2.1.-2.2.	94.7	31.7	165	18.4	106.5	18.4	97	27.1
	2.2.-2.3.	195.6	28.3	221	39.4	265.6	39.4	158.3	31.9
4	*1.1.-1.2.*	148.9	25.1	85.6	20.6	53.2	14.9	101.3	23.7
	1.2.-1.3.	160.3	28.5	53.8	16.5	46.9	14.2	115.2	20.1
	2.1.-2.2.	97.6	14	158.4	25.4	132.1	21.1	161	22.6

	2.2.-2.3.	264	41.2	290.9	33.6	386.9	46.3	380.8	53
5	*1.1.-1.2.*	81.7	18.2	104	25.7	102.1	25.3	89.5	19
	1.2.-1.3.	55.6	17.7	124	34	178.7	29.4	75.2	23
	2.1.-2.2.	18.8	3.6	230	52.6	299.2	60.3	63.4	21.9
	2.2.-2.3.	73.1	25.5	124.1	55.8	292	74.8	91.7	22.6
6	*1.1.-1.2.*	64.6	9.8	122	19.8	217.9	40.1	131.3	23.1
	1.2.-1.3.	60.5	7	66	11.3	255.1	24.2	81	11.4
	2.1.-2.2.	29.1	4.5	53.8	7.9	161.5	39.5	57.7	8.4
	2.2.-2.3.	21.1	2.9	52.9	9.8	109.2	17.1	61.8	10
7	*1.1.-1.2.*	178.2	31.3	241.3	31.3	307.6	40.9	277.6	41.5
	1.2.-1.3.	84.9	18.9	169.4	29.8	214.8	34.6	189.5	38.1
	2.1.-2.2.	57.1	17.9	122.3	26.2	185.7	29.6	175.1	37.9
	2.2.-2.3.	122.8	24.2	179.3	28.3	280.7	28.5	198.4	34.8
8	*1.1.-1.2.*	87.8	14.6	121.7	20	174.3	21.5	167.3	25.8
	1.2.-1.3.	144.3	32.1	170.1	27.5	210.8	27.7	197.6	32.6
	2.1.-2.2.	121.3	28.7	144.3	21.2	200.1	41.2	190.3	35.7
	2.2.-2.3.	97.3	23.7	134.6	54.7	190.4	27.5	180.9	33.2
9	*1.1.-1.2.*	231.5	28.9	204.5	34.4	266.5	43.3	190.8	34.4
	1.2.-1.3.	339.4	20.5	295.9	32.3	347.9	39.7	207.6	26.5
	2.1.-2.2.	393.6	21.9	437.6	31	495.9	88.4	322.4	40.3
	2.2.-2.3.	323.7	20.6	366.2	22.1	390.7	21.9	293.3	19.4
10	*1.1.-1.2.*	117.9	33.7	132.5	52.1	181.6	27.1	118.4	21.8
	1.2.-1.3.	381.2	40.2	398.6	44.4	415.6	51.8	317	47.4
	2.1.-2.2.	75.4	23.1	84.9	20.4	92.1	25.6	76.3	23.2
	2.2.-2.3.	163.9	37	188.7	45.9	223	31.1	173	33.6
11	*1.1.-1.2.*	153	29.4	201.5	37.5	304.7	29.5	243.1	27.8
	1.2.-1.3.	99.6	30.9	189.2	39.2	300.4	30.9	187.1	24.3
	2.1.-2.2.	101.4	49.1	236.1	40.1	381.9	49.1	261.1	32.6
	2.2.-2.3.	80.9	30.3	134.5	24.3	216.1	30.3	218.1	24.6
12	*1.1.-1.2.*	25.2	6.8	27.6	5.2	87.2	27.4	85.6	25.2
	1.2.-1.3.	14.4	6	21	4.7	63.1	15.7	60.9	21.1
	2.1.-2.2.	168.2	25.2	195.5	27.8	230	27.2	211.3	26.8
	2.2.-2.3.	12.6	3.3	15.1	1.8	59.3	13.8	54.1	11.1
